# Phylogenetic Distribution and Predicted Functional and Ecological Shifts in Soil Bacterial Communities Along a Soda Saline–Alkali Wetland Degradation Gradient

**DOI:** 10.3390/life16050760

**Published:** 2026-05-01

**Authors:** Junnan Ding, Xue Cong, Xin Li

**Affiliations:** 1 Heilongjiang Province Key Laboratory of Cold Region Wetland Ecology and Environment Research, Harbin University, Harbin 150086, China; cxmails@163.com; 2 College of Resources and Environment, Northeast Agricultural University, Harbin 150030, China; swx05256lx@126.com

**Keywords:** wetland degradation, soda saline–alkali wetland, soil bacterial communities, phylogenetic distribution, predicted functional profiles

## Abstract

Wetland degradation in soda saline–alkali ecosystems can profoundly alter belowground microbial communities, yet its effects on bacterial phylogenetic distribution and predicted ecological characteristics remain insufficiently understood. This study investigated soil physicochemical properties, enzyme activities, and bacterial communities across a wetland degradation gradient in the Halahai Provincial Nature Reserve, China, including reed wetland (RW), meadow steppe (MS), and degraded *Suaeda* saline patches (DS). Soil analyses were integrated with 16S rRNA gene amplicon sequencing, phylogenetic reconstruction, and FAPROTAX and BugBase prediction. DS showed significantly higher pH and electrical conductivity, but lower soil water content, organic carbon, nutrient availability, and urease activity than RW and MS. Alpha diversity analysis indicated that DS had lower bacterial richness and diversity, but higher dominance, whereas RW and MS did not differ significantly. Beta-diversity analysis revealed clear habitat-dependent separation, with DS harboring the most distinct community structure. Taxonomic and phylogenetic analyses indicated enrichment of *Gemmatimonadota* and the RCP2-54 lineage in DS, whereas RW and MS were more strongly associated with *Pseudomonadota*, *Acidobacteriota*, and related groups. Predicted functional and phenotypic analyses further suggested a shift toward stress-related and degradation-associated traits in DS. These findings demonstrate that wetland degradation reshaped the taxonomic composition, phylogenetic distribution, and predicted ecological characteristics of soil bacterial communities in this fragile ecosystem.

## 1. Introduction

Wetlands are among the most important ecosystems on Earth, playing essential roles in global biogeochemical cycling, biodiversity conservation, and climate regulation [1]. However, these ecosystems are highly sensitive to environmental change, particularly in regions where hydrological instability and salinization accelerate habitat degradation [2]. Soil salinization and alkalization have become major global environmental constraints, affecting approximately 10% of the world’s arable land [3]. Saline soils are commonly characterized by electrical conductivity values exceeding 4 dS·m^−1^, whereas alkaline or sodic soils frequently exhibit pH values above 8.5, conditions that can strongly restrict plant growth and alter belowground biological processes [4]. The Songnen Plain in Northeast China is one of the world’s largest soda saline–alkali areas and is characterized by high concentrations of Na_2_CO_3_ and NaHCO_3_, elevated soil pH, and poor soil structural stability [5]. Under the combined effects of environmental fluctuation and habitat degradation, parts of this region have undergone pronounced transitions from natural *Phragmites australis* wetlands to meadow steppe and severely degraded *Suaeda glauca*-dominated saline patches [6]. Such habitat transitions provide an ideal natural gradient for investigating how wetland degradation reshapes belowground bacterial communities in soda saline–alkali ecosystems [7].

Soil bacteria are crucial for organic matter decomposition, nutrient cycling, and overall ecosystem functioning in wetlands [8]. In soda saline–alkali soils, bacterial community composition and activity are strongly influenced by edaphic factors, particularly high pH, salinity, and nutrient limitation, which act as key environmental filters shaping community assembly [9]. Soil extracellular enzymes, including urease, alkaline phosphatase, sucrase, and catalase, play essential roles in substrate decomposition and nutrient transformation, and are commonly used as indicators of soil biochemical functioning and microbial metabolic activity [10,11]. However, it is important to recognize that these enzymes serve distinct functions. Urease and alkaline phosphatase are involved in nitrogen and phosphorus transformations, respectively, while sucrase specifically catalyzes the breakdown of sucrose and is not a standard indicator in saline–alkali soils [12]. Catalase is an oxidoreductase enzyme that is primarily involved in oxidative stress responses rather than nutrient transformation [13]. Previous research has documented taxonomic shifts in microbial communities across saline or degraded soils, but the relationship between bacterial community variation and changes in soil enzyme activity and edaphic conditions during soda saline–alkali wetland degradation remains insufficiently understood [14].

Most previous investigations of soil microbiomes have focused primarily on taxonomic composition, whereas comparatively less attention has been paid to the phylogenetic and functional dimensions of community response [15]. In extreme environments, environmental filtering may not only alter the relative abundance of taxa, but may also favor bacterial lineages with conserved stress-tolerant traits [16]. In soda saline–alkali wetlands, elevated pH and salinity are expected to act as major environmental filters on bacterial community composition, thereby influencing the distribution of dominant bacterial lineages and their associated ecological traits [17]. Previous studies in saline or degraded wetland soils have primarily focused on shifts in microbial taxonomic composition and their relationships with soil physicochemical properties and enzyme activities [18]. More recent work has also begun to examine community assembly patterns in saline–alkali wetland degradation [19]. However, few studies have jointly integrated bacterial taxonomic composition, phylogenetic distribution, predicted functional profiles, and ecological phenotypes along a soda saline–alkali wetland degradation gradient [20]. While previous research has examined these factors individually, the combined use of phylogenetic reconstruction with FAPROTAX and BugBase prediction remains limited in this ecosystem type [21]. Specifically, studies that integrate these different dimensions, taxonomic diversity, functional prediction, and phenotypic profiling, remain rare, and this gap highlights the novelty of our approach in providing a multidimensional perspective of bacterial community dynamics across a degradation gradient [22]. Consequently, the ecological links among edaphic variation, bacterial lineage turnover, and predicted functional change in these fragile ecosystems are still poorly resolved.

To address this knowledge gap, we investigated soil properties, enzyme activities, and bacterial communities in the Halahai Provincial Nature Reserve, Heilongjiang Province, China, across three representative habitat stages along a wetland degradation gradient: reed wetland (RW), meadow steppe (MS), and degraded *Suaeda* saline patches (DS). By integrating soil physicochemical and enzymatic analyses with high-throughput 16S rRNA gene sequencing, phylogenetic reconstruction of dominant bacterial lineages, and FAPROTAX and BugBase prediction, this study provides a multidimensional framework for understanding bacterial responses to wetland degradation in soda saline–alkali wetlands. Specifically, we aimed to: (1) characterize variations in soil physicochemical properties and enzyme activities across the soda saline–alkali wetland degradation gradient; (2) determine how bacterial diversity, taxonomic composition, community structure, and phylogenetic distribution respond to habitat transition; and (3) evaluate shifts in predicted functional profiles and ecological phenotypes, together with their major environmental correlates. We hypothesized that wetland degradation would intensify salinity–alkalinity stress and reduce nutrient availability, especially in the degraded *Suaeda* habitat, thereby promoting a more distinct bacterial community structure than in RW and MS. We further expected that degraded habitats would be enriched in stress-associated dominant lineages, whereas wetter and less degraded habitats would retain bacterial groups more closely associated with relatively favorable moisture and nutrient conditions. Correspondingly, we predicted that these lineage-level shifts would be accompanied by increases in stress-related and degradation-associated predicted functional profiles and ecological phenotypes in DS. By linking edaphic variation, bacterial community reassembly, phylogenetic distribution, and predicted ecological potential, this study provides new insight into the microbial ecological consequences of degradation in soda saline–alkali wetlands.

## 2. Materials and Methods

### 2.1. Study Area

**Figure 1:**
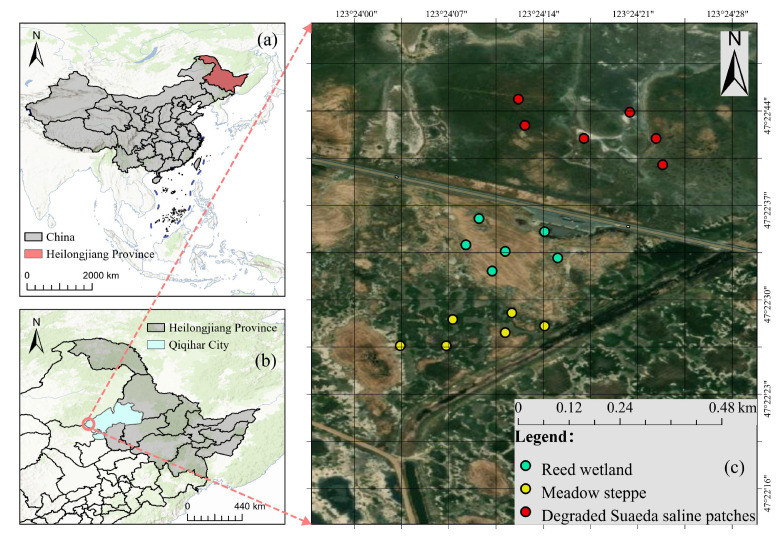
Location of the study area and distribution of sampling habitats. Note: (**a**) Location of Heilongjiang Province in China; (**b**) location of Qiqihar City in Heilongjiang Province; (**c**) spatial distribution of sampling plots representing reed wetland, meadow steppe, and degraded *Suaeda* saline patches in the Halahai Provincial Nature Reserve.

The study was conducted in the Halahai Provincial Nature Reserve (47°34′ N, 124°32′ E), situated within Qiqihar City, Heilongjiang Province, Northeast China. Geographically, the reserve is located in the lower reaches of the Wuyuer River, a significant endorheic river that terminates in expansive marshes and serves as a primary upstream water source for the Zhalong National Nature Reserve. This region lies in the transitional zone of the semi-arid Songnen Plain, which is recognized globally as one of the three largest soda saline–alkali distribution areas [15]. The local climate is a typical temperate continental monsoon type, with a mean annual temperature of 2.2 °C and a frost-free period of approximately 130 days. Precipitation is relatively scarce, with a mean annual total of 427.4 mm, more than 70% of which is concentrated during the growing season from June to August. Notably, the potential evaporation rates (1300–1600 mm·year^−1^) significantly exceed precipitation. The geographic location of the study area and the distribution of the sampling habitats are shown in Figure 1.

### 2.2. Sampling Sites and Experimental Design

To examine how wetland degradation influences soil physicochemical properties and bacterial community assembly in a soda saline–alkali ecosystem, three representative habitat types along a natural degradation gradient were selected in the Halahai Provincial Nature Reserve. These habitats differed markedly in hydrological conditions, vegetation composition, and salinity–alkalinity status. The RW represented the least disturbed wetland habitat, maintaining relatively high soil moisture and seasonal or prolonged surface water retention. This habitat was dominated by typical hygrophytic vegetation, including *Phragmites australis*, *Typha angustifolia*, and *Echinochloa crusgalli*. The MS represented a transitional habitat between the wetland and adjacent terrestrial grassland, where reduced waterlogging and moderate habitat drying supported the establishment of mesophytic species such as *Leymus chinensis*, *Calamagrostis epigeios*, *Setaria viridis*, and *Scirpus triqueter*. The DS represented a severely degraded saline–alkali habitat, characterized by weakened hydrological connectivity, surface salt accumulation, and sparse halophytic vegetation. This habitat was mainly dominated by *Suaeda glauca* and *Puccinellia tenuiflora*, with occasional occurrence of *Pennisetum alopecuroides*. At each habitat type, six spatially independent plots (10 m × 10 m) were established as biological replicates, with sufficient distance between plots to ensure sampling independence. All plots were selected from relatively homogeneous areas within each habitat type to minimize the influence of microsite heterogeneity on soil and microbial measurements.

### 2.3. Soil Sampling and Processing

Soil sampling was conducted from 6 to 8 October 2025. In each of the three habitat types, namely RW, MS, and DS, six spatially independent plots (10 m × 10 m) were established as biological replicates. Within each plot, soil samples were collected from the 0–20 cm layer using a sterile stainless steel soil auger (5 cm in diameter) following a standard five-point sampling scheme, including the center and four corners of each plot. To avoid cross-contamination, the auger was cleaned and sterilized with 75% ethanol between plots. The five soil cores collected from each plot were thoroughly homogenized to form one composite sample, thereby reducing microsite heterogeneity. Visible plant roots, stones, and coarse debris were removed manually. In total, 18 composite soil samples were obtained (3 habitat types × 6 replicates). Each composite sample was then divided into three subsamples for subsequent analyses. One subsample was transferred into sterile centrifuge tubes, immediately frozen in liquid nitrogen in the field, and stored at −80 °C for genomic DNA extraction. A second subsample was stored at 4 °C in portable coolers and analyzed within one week for the determination of soil enzyme activities and selected available nutrient indices. The remaining subsample was air-dried at room temperature, gently ground after removal of visible roots and debris, and passed through a 2 mm sieve for the determination of soil pH, electrical conductivity, and available nutrients, and through a 0.149 mm sieve for the analysis of soil organic carbon and total nitrogen.

### 2.4. Soil Physicochemical Analysis

Soil water content (SWC) was determined gravimetrically by drying fresh soil at 105 °C to a constant weight [23]. Soil pH was measured in a 1:2.5 (*w*/*v*) soil-to-water suspension using a digital pH meter (Mettler Toledo SevenCompact S220, Mettler Toledo, Greifensee, Switzerland) [24], whereas electrical conductivity (EC) was determined in a 1:5 (*w*/*v*) soil-to-water extract using a conductivity meter (Thermo Fisher Scientific, Waltham, MA, USA) [25]. The different extraction ratios were selected according to standard analytical practice, with 1:2.5 commonly used for stable pH determination and 1:5 widely adopted for estimating soluble salt content and salinity in saline–alkali soils. Soil organic carbon (SOC) was quantified by the potassium dichromate oxidation with external heating method (Merck KGaA, Darmstadt, Germany) and total nitrogen (TN) was determined using the semi-micro Kjeldahl digestion method (Fisher Scientific, Waltham, MA, USA) [26]. Total phosphorus (TP) was digested with H_2_SO_4_-HClO_4_ and determined by the molybdenum–antimony colorimetric method (Fisher Scientific, Waltham, MA, USA) [27]. Total potassium (TK) was measured after NaOH fusion digestion using flame photometry (Sherwood Scientific Ltd., Cambridge, UK) [28]. Available nitrogen (AN, alkali-hydrolyzable nitrogen) was determined by the alkaline hydrolysis diffusion method (Sigma-Aldrich, St. Louis, MO, USA) [29]. Available phosphorus (AP) was extracted with 0.5 mol·L^−1^ NaHCO_3_ (pH 8.5) and determined colorimetrically (Thermo Fisher Scientific, Waltham, MA, USA) [30], while available potassium (AK) was extracted with 1.0 mol·L^−1^ NH_4_OAc (pH 7.0) and measured by flame photometry (Sherwood Scientific Ltd., Cambridge, UK) [31].

### 2.5. Soil Enzyme Activity Assays

Soil urease (URE) activity was determined following the conventional colorimetric procedure described by Kandeler and Gerber [32]. Fresh soil was incubated with 10% urea solution at 37 °C for 24 h, and the released ammonium was quantified using the indophenol blue colorimetric method at 578 nm. URE activity was expressed as mg NH_3_-N g^−1^ soil 24 h^−1^. Soil alkaline phosphatase (ALP) activity was assayed by incubating soil with disodium phenyl phosphate solution in borate buffer (pH 9.6) at 37 °C for 24 h, and the released phenol was determined colorimetrically at 510 nm using 4-aminoantipyrine [33]. ALP activity was expressed as mg phenol g^−1^ soil 24 h^−1^. Soil sucrase (SUC) activity was measured by incubating soil with 8% sucrose solution at 37 °C for 24 h. The reducing sugars released were reacted with 3,5-dinitrosalicylic acid (DNS), and absorbance was measured at 508 nm [34]. SUC activity was expressed as mg glucose g^−1^ soil 24 h^−1^. Soil catalase (CAT) activity was determined by reaction with 0.1 mol·L^−1^ H_2_O_2_, and the residual H_2_O_2_ was quantified by titration with 0.1 mol·L^−1^ KMnO_4_ [35]. CAT activity was expressed as mL 0.1 mol·L^−1^ KMnO_4_ g^−1^ soil 20 min^−1^ [36].

### 2.6. DNA Extraction and High-Throughput 16S rRNA Gene Paired-End Sequencing

Total genomic DNA was extracted from approximately 0.5 g of fresh soil using the E.Z.N.A.^®^ Soil DNA Kit (Omega Bio-tek, Norcross, GA, USA) according to the manufacturer’s instructions. DNA quality was assessed using a NanoDrop 2000 spectrophotometer (Thermo Fisher Scientific, Waltham, MA, USA) and 1% agarose gel electrophoresis. The V3–V4 region of the bacterial 16S rRNA gene was amplified using primers 338F and 806R [37]. PCR was performed with an initial denaturation at 95 °C for 3 min, followed by 27 cycles of 95 °C for 30 s, 55 °C for 30 s, and 72 °C for 45 s, and a final extension at 72 °C for 10 min. PCR products were purified using the AxyPrep DNA Gel Extraction Kit (Axygen Scientific, Union City, CA, USA) [34], quantified with a Quantus™ Fluorometer (Promega, Madison, WI, USA), pooled in equimolar amounts, and sequenced on the Illumina NovaSeq 6000 platform (2 × 250 bp) at Majorbio Bio-Pharm Technology Co., Ltd. (Shanghai, China). Raw sequence data have been deposited in the NCBI Sequence Read Archive under BioProject accession number PRJNA1438915.

### 2.7. Bacterial Community Statistical and Bioinformatic Analyses

Raw sequencing data were processed on the Majorbio Cloud Platform. After quality filtering and merging, reads were clustered de novo into operational taxonomic units (OTUs) at 97% similarity using UPARSE (v7.1) [38], and taxonomic assignment was performed against the SILVA 16S rRNA database (release 138) using the RDP Classifier (v2.11) [39]. To normalize sequencing depth, all samples were rarefied to the minimum sequencing depth. All statistical analyses were performed in R (v4.2.0). Alpha diversity indices (Sobs, Chao1, Shannon, and Simpson) were compared among habitat types using one-way ANOVA followed by Tukey’s HSD test. Shared and unique OTUs were visualized using Venn diagrams, and differentially abundant taxa were identified using LEfSe (LDA > 4.0) [40]. Beta-diversity was evaluated using Bray–Curtis distance-based PCoA and NMDS in the vegan package (v2.6-4), and differences among habitat types were tested by ANOSIM with 999 permutations [41]. A maximum-likelihood phylogenetic tree of dominant taxa was constructed in MEGA (v11.0). Relationships between bacterial communities and environmental factors were analyzed using RDA, envfit, Mantel tests, and Spearman correlation analysis. Functional profiles and ecological phenotypes were predicted using FAPROTAX (v1.2.7) [42] and BugBase [43], respectively.

### 2.8. Statistical Analysis

For alpha diversity analysis, the Sobs, Chao1, Shannon, and Simpson indices were compared among habitat types using the Kruskal–Wallis test. To account for multiple testing, *p*-values were adjusted using the false discovery rate (FDR) method, and post hoc pairwise comparisons were performed using the Tukey–Kramer test. The corresponding H statistic and exact *p*-value were reported for each index. Data in tables are presented as mean ± standard deviation unless otherwise stated. Statistical significance was defined at *p* < 0.05.

## 3. Results

### 3.1. Changes in Soil Physicochemical Properties and Enzyme Activities Along the Wetland Degradation Gradient

**Figure 2:**
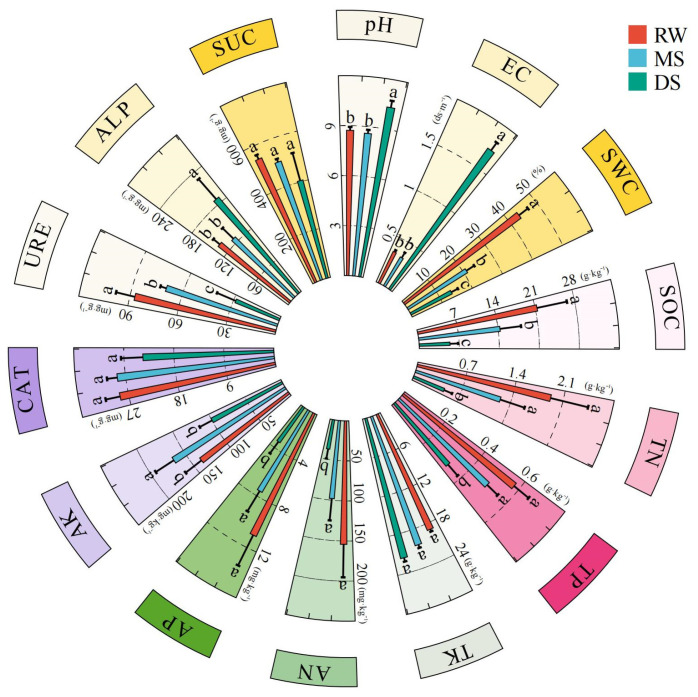
Changes in soil physicochemical properties and enzyme activities along the wetland degradation gradient. Note: Data are presented as the mean ± standard error (*n* = 6). Different lowercase letters indicate significant differences among habitat types according to Tukey’s HSD test at *p* < 0.05. Abbreviations: pH, soil pH; EC, electrical conductivity; SWC, soil water content; SOC, soil organic carbon; TN, total nitrogen; TP, total phosphorus; TK, total potassium; AN, available nitrogen; AP, available phosphorus; AK, available potassium; URE, urease activity; ALP, alkaline phosphatase activity; SUC, sucrase activity; CAT, catalase activity; RW, reed wetland; MS, meadow steppe; DS, degraded *Suaeda* patch.

As shown in Figure 2, soil physicochemical properties and enzyme activities differed significantly among the three habitat types. Soil pH and EC were significantly higher in DS than in RW and MS (*p* < 0.05), indicating stronger salinity–alkalinity stress in the degraded habitat. In contrast, SWC, SOC, and URE were highest in RW (*p* < 0.05). TN, TP, AN, and AP were significantly higher in RW and MS than in DS (*p* < 0.05), whereas AK was highest in MS, exceeding that in RW and DS by 16.85% and 40.05%, respectively (*p* < 0.05). No significant differences were observed in TK, CAT, or SUC among habitat types.

### 3.2. Alpha Diversity of Bacterial Communities Across Habitat Types

As shown in Table 1, bacterial alpha diversity differed significantly among the three habitat types according to the Kruskal–Wallis test. The Sobs and Chao1 indices showed similar patterns, with DS exhibiting significantly lower values than RW and MS, whereas no significant difference was detected between RW and MS in the post hoc comparisons. Specifically, the overall differences were significant for Sobs (H = 11.47, *p* = 0.00323) and Chao1 (H = 11.41, *p* = 0.00332). A similar trend was observed for the Shannon index, which was also significantly lower in DS than in RW and MS (H = 9.55, *p* = 0.00842). In contrast, the Simpson index was significantly higher in DS than in RW and MS, while RW and MS again did not differ significantly from each other (H = 9.69, *p* = 0.00784).

### 3.3. Compositional Characteristics and Differential Abundance Analysis of Soil Microbial Communities

**Figure 3:**
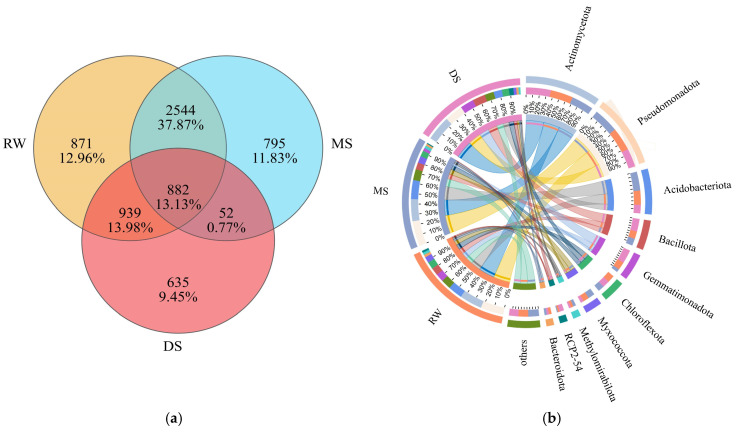
Shared and unique OTUs (**a**) and phylum-level composition of soil bacterial communities (**b**) along the wetland degradation gradient.

**Figure 4:**
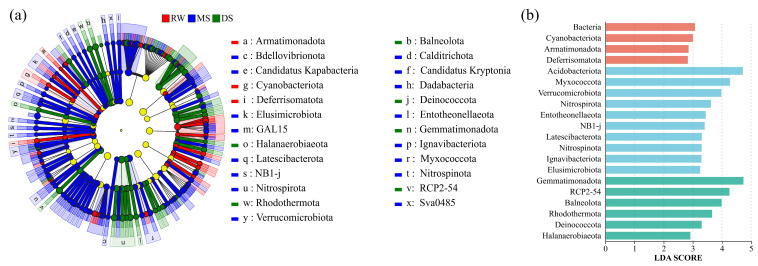
LEfSe analysis identifying differentially abundant bacterial taxa among the three habitat types. (**a**) Cladogram showing the taxonomic distribution of differentially abundant bacterial taxa from the phylum to genus level; (**b**) LDA score distribution of differentially abundant taxa (LDA > 4.0).

As shown in the Venn diagram (Figure 3a), a total of 6718 OTUs were identified across the three habitat types, of which 882 (13.13%) were shared among RW, MS, and DS as core bacterial OTUs. At the phylum level (Figure 3b), the bacterial communities in all three habitat types were predominantly composed of *Actinomycetota*, *Pseudomonadota*, *Acidobacteriota*, and *Gemmatimonadota*. LEfSe analysis (Figure 4; LDA > 4.0) further identified distinct bacterial biomarkers among habitat types. The cladogram (Figure 4a) and LDA score distribution (Figure 4b) showed that *Pseudomonadota*, especially *Alphaproteobacteria* and *Sphingomonadales*, were significantly enriched in RW. In MS, the main discriminant taxa were *Acidobacteriota* (LDA = 4.72) and *Myxococcota* (LDA = 4.28), together with specific lineages within *Actinomycetota*, including *Micrococcales* and *Arthrobacter*. In contrast, DS was mainly characterized by the enrichment of *Gemmatimonadota* (LDA = 4.74) and the RCP2-54 lineage (LDA = 4.26).

### 3.4. Beta-Diversity Analysis of Soil Microbial Community Structure

**Figure 5:**
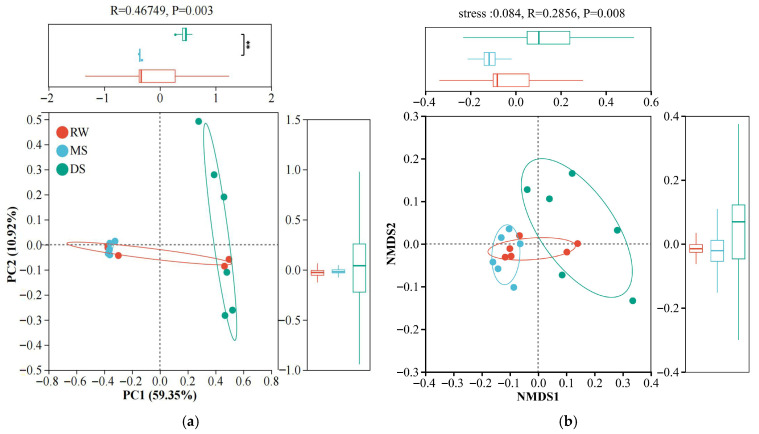
Principal coordinate analysis (PCoA) and non-metric multidimensional scaling (NMDS) of soil bacterial communities along the wetland degradation gradient based on Bray–Curtis distances. (**a**) PCoA ordination showing the distribution of bacterial community composition among habitat types; (**b**) NMDS ordination showing differences in bacterial community structure among habitat types. ** represents *p* < 0.01, and lines indicate significant comparisons between groups.

Principal coordinate analysis (PCoA, Figure 5a) and non-metric multidimensional scaling (NMDS, Figure 5b) were performed to assess differences in bacterial community structure among the three habitat types. In the PCoA ordination, the first two axes explained 59.35% and 10.92% of the total variation, respectively, accounting for 70.27% cumulatively. The samples from RW, MS, and DS formed distinct clusters, with clear separation mainly along the first principal coordinate. NMDS analysis showed a pattern highly consistent with the PCoA result, and the low stress value (0.084) indicated that the ordination provided a reliable representation of community dissimilarities among samples. In the NMDS plot, RW and MS samples were positioned relatively close to each other, whereas DS samples were clearly separated and formed an independent cluster. The statistical test further confirmed significant differences in bacterial community structure among habitat types (R = 0.2856, *p* = 0.008), indicating that DS harbored the most distinct bacterial community structure.

### 3.5. Phylogenetic Relationships of Dominant Bacterial Lineages Across Habitat Types

**Figure 6:**
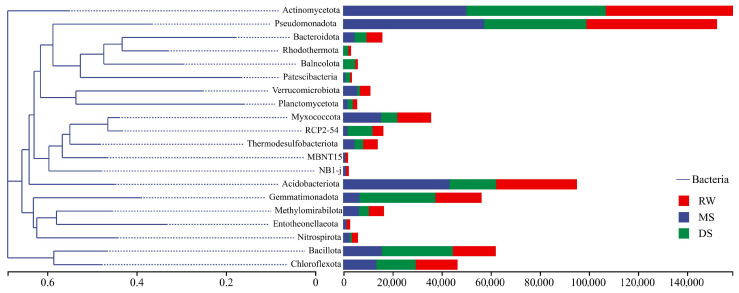
Maximum-likelihood phylogenetic relationships of dominant bacterial phyla across the three habitat types. Note: The bar plots surrounding the tree represent the relative abundance of each phylum in RW, MS, and DS.

A maximum-likelihood phylogenetic tree was constructed to provide a descriptive overview of the relative phylogenetic relationships among the dominant bacterial phyla (Figure 6). The tree showed that these taxa were distributed across several major phylogenetic branches. *Pseudomonadota*, *Actinomycetota*, and *Bacteroidota* occurred within one major branch and showed relatively high abundances in RW and MS. *Gemmatimonadota*, *Acidobacteriota*, and the RCP2-54 lineage were located within another branch and were comparatively more abundant in DS. In addition, *Myxococcota*, *Planctomycetota*, and *Verrucomicrobiota* formed another cluster, with relatively higher abundances in MS. These patterns provide a descriptive phylogenetic context for the habitat-associated distribution of dominant bacterial lineages, but they should not be interpreted as direct evidence of community assembly mechanisms.

### 3.6. Relationships Between Bacterial Communities and Environmental Factors

**Figure 7:**
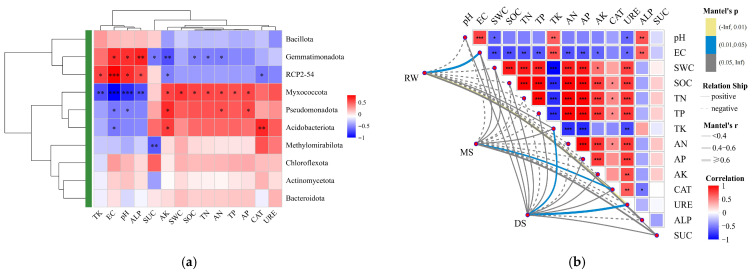
Relationships between soil environmental factors and bacterial communities. (**a**) Mantel test showing the relationships between bacterial community variation and soil environmental factors; (**b**) Spearman correlation heatmap showing the associations between dominant bacterial phyla and environmental variables; red and blue colors indicate positive and negative correlations, respectively. * *p* < 0.05, ** *p* < 0.01, *** *p* < 0.001.

To explore the relationships between bacterial community variation and soil environmental factors, Mantel tests, Spearman correlation analysis, and redundancy analysis (RDA) combined with envfit tests were performed (Table S1). Mantel analysis showed that bacterial community variation was significantly correlated with total potassium (TK, *r* = 0.482, *p* < 0.05) and urease activity (URE, *r* = 0.625, *p* < 0.05) (Figure 7a). RDA and envfit analysis further indicated that pH, EC, and available potassium (AK) were important factors associated with community variation (Table S1). At the taxonomic level, Spearman correlation analysis (Figure 7b) revealed differentiated responses of dominant bacterial phyla to environmental variables, with *Pseudomonadota* and *Acidobacteriota* showing significant positive correlations with AK (*p* < 0.01).

### 3.7. Habitat-Dependent Variation in Predicted Functional Profiles of Soil Bacterial Communities

**Figure 8:**
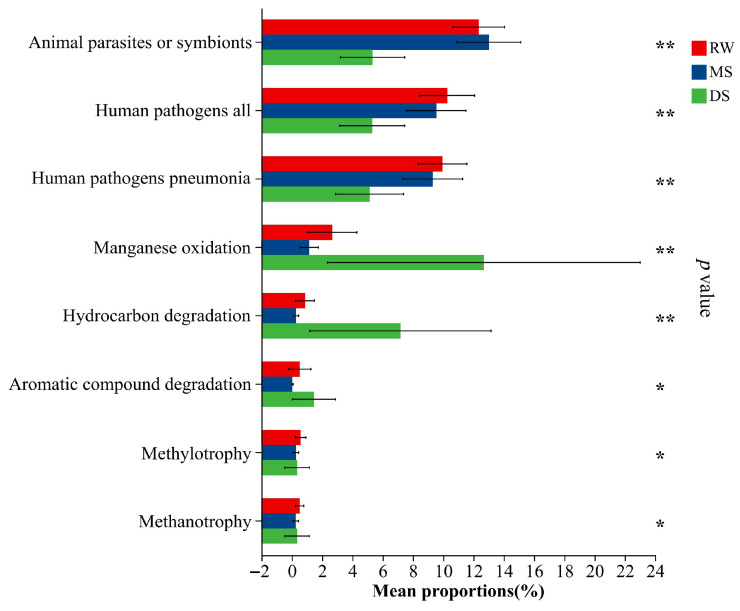
Predicted functional shifts in soil bacterial communities along the wetland degradation gradient. Note: significance levels: * *p* < 0.05, ** *p* < 0.01 after FDR correction.

FAPROTAX-based prediction indicated significant habitat-dependent variation in the potential functional structure of soil bacterial communities (Figure 8). After multiple-testing correction, five functional groups remained significantly different among habitat types (adjusted *p* < 0.05). Functions related to animal parasites or symbionts, human pathogens all, and human pathogens pneumonia showed higher relative abundances in RW and MS than in DS, whereas manganese oxidation and hydrocarbon degradation were more abundant in DS. Although aromatic compound degradation, methylotrophy, and methanotrophy also varied among habitat types at the nominal level, these differences were not significant after false discovery rate correction. These results should be interpreted as predicted functional tendencies inferred from taxonomic composition rather than as direct measurements of in situ metabolic activity.

### 3.8. Habitat-Dependent Variation in Predicted Ecological Phenotypes of Soil Bacterial Communities

**Figure 9:**
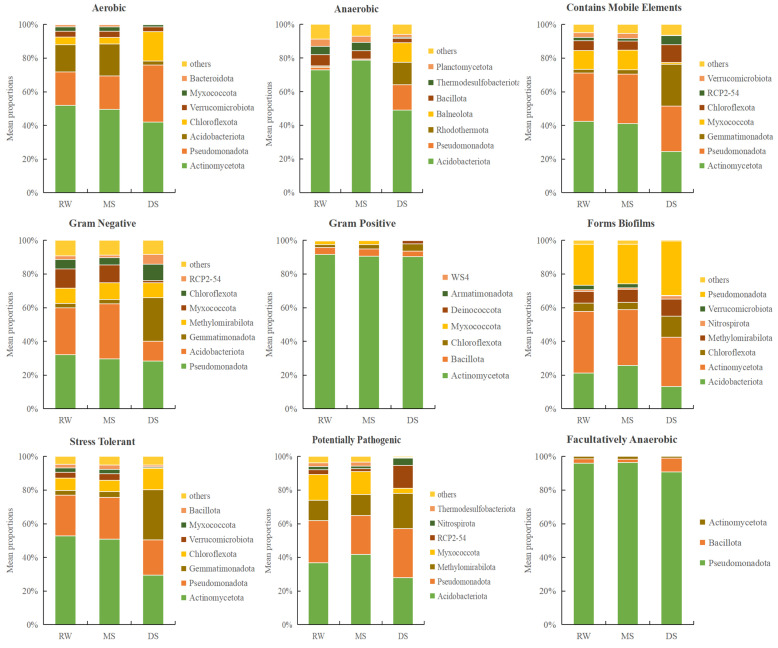
Phylum-level distribution of BugBase-predicted ecological phenotypes across habitat types.

BugBase-based phenotype prediction further indicated habitat-dependent variation in the phylum-level distribution of predicted ecological phenotypes (Figure 9). *Actinomycetota* was the dominant contributor to several predicted phenotypes, including aerobic, Gram-positive, forms biofilms, contains mobile elements, and stress-tolerant, especially in RW and MS. *Pseudomonadota* showed consistently high contributions to Gram-negative and facultatively anaerobic phenotypes and remained important in biofilm formation- and mobile element-related categories. *Acidobacteriota* mainly contributed to the anaerobic phenotype across all habitat types. In contrast, *Gemmatimonadota* made a marked contribution to stress-tolerant, Gram-negative, and mobile element-related phenotypes in DS, suggesting a stronger association with the degraded saline–alkali habitat. These results indicate that wetland degradation was accompanied by shifts in the predicted ecological phenotype distribution of dominant bacterial phyla, although such predictions should be interpreted cautiously in soil environments.

## 4. Discussion

Wetland degradation across the RW–MS–DS gradient was accompanied by a marked transition in soil physicochemical conditions, with DS showing elevated pH and EC but reduced SWC, SOC, TN, TP, AN, and AP relative to RW and MS. This pattern indicates that degradation in the soda saline–alkali wetland intensified salinity–alkalinity stress while simultaneously weakening water and nutrient supply, which is consistent with the general view that hydrological disconnection and surface salt accumulation are central drivers of soil deterioration in degraded wetlands [44,45,46]. The decline in urease activity toward DS further suggests that nitrogen transformation potential was constrained under stronger alkaline and osmotic stress, and also indicates that microbial N acquisition and ammonification-related processes became less active in the degraded habitat [47]. At the same time, because urease activity assays in highly alkaline soils may be influenced by abiotic urea hydrolysis, the URE results in this study should be interpreted primarily as comparative indicators among habitat types rather than as absolute estimates of in situ N transformation rates [48]. Soil extracellular enzymes function as key mediators of nutrient turnover, and their activities can therefore provide a biochemical reflection of microbial metabolic demand and resource acquisition strategies under changing environmental conditions [49]. In contrast, the relatively stable CAT and SUC activities imply that not all enzymatic processes respond to degradation with the same sensitivity. This differential response suggests that saline–alkali stress may exert stronger constraints on N-related biochemical processes than on general oxidative protection or carbon substrate decomposition, although these processes remain influenced by microbial adaptation and substrate availability [50]. Similar enzyme-specific responses have been linked to differences in substrate limitation, microbial demand, and the direct effects of salinity and pH on extracellular enzyme stability and activity [51]. Taken together, the observed enzyme patterns indicate that wetland degradation altered not only the soil chemical environment but also the biochemical pathways through which microbial communities access and transform nutrients, thereby contributing to the habitat-dependent reassembly of bacterial communities [52]. In this context, the environmental contrast between RW and DS provides the key ecological background for understanding the subsequent shifts in bacterial communities.

The alpha diversity results indicate that wetland degradation was not associated with a simple linear response across habitat types. In the present study, the DS showed significantly lower Sobs, Chao1, and Shannon indices than RW and MS, whereas no significant differences were found between RW and MS. This pattern suggests that the most significant decline in bacterial richness and diversity occurred only under the more severe saline–alkali conditions of DS, where elevated pH and EC, together with reduced water and nutrient availability, likely intensified environmental filtering [53]. Under such stressful conditions, only a narrower subset of bacterial taxa with greater tolerance to saline–alkali stress may persist, resulting in reduced richness and diversity [54]. Consistent with this interpretation, the Simpson index was significantly higher in DS, indicating greater dominance and a more uneven community structure in the degraded habitat [55]. Comparable declines in microbial diversity under intensified salinity or degradation stress have been reported in saline–alkali soils and degraded wetlands, where strong abiotic filtering limits the persistence of sensitive taxa and promotes the dominance of a smaller number of adapted groups [56,57]. By contrast, the relatively lower diversity in RW may reflect stronger environmental filtering imposed by prolonged soil moisture and reduced oxygen availability [58]. Taxonomic analyses support this interpretation, as RW and MS shared more OTUs and exhibited greater community similarity than MS and DS, while LEfSe further revealed clear habitat-associated biomarkers. The enrichment of *Pseudomonadota* in RW may be related to the relatively moist and resource-rich wetland conditions, which can support multiple bacterial groups involved in aerobic and facultatively anaerobic processes, rhizosphere-associated interactions, and rapid substrate utilization, rather than reflecting a generic ecological flexibility of the phylum itself. By contrast, the enrichment of *Acidobacteriota* and *Myxococcota* in MS may indicate adaptation to intermediate moisture and resource conditions [59,60]. These habitat-associated enrichment patterns describe differences among land-use types, but they do not necessarily imply one-to-one correspondence with correlations observed across all environmental variables at the whole-dataset level. The strong enrichment of *Gemmatimonadota* and the RCP2-54 lineage in DS is ecologically meaningful because DS represented the most stressful habitat along the degradation gradient, with higher salinity–alkalinity stress and lower nutrient availability than RW and MS [61]. Previous studies have shown that *Gemmatimonadota* and related lineages are frequently associated with dry, nutrient-limited, and stress-prone soils, suggesting that their enrichment in DS reflects stronger environmental filtering and the selection of bacterial groups better adapted to intensified saline–alkali stress [62,63]. These taxa are known for possessing traits such as osmoprotection, carbonate tolerance, and alkaliphily, which allow them to thrive under such conditions. However, it is important to note that the 16S rRNA gene relative abundance data, which reflect community composition, cannot directly demonstrate functional adaptation or ecological strategies of the bacterial groups. 16S relative abundance only provides information about the taxonomic distribution of microbial communities and does not directly correlate with microbial function or adaptive traits. Thus, while we have observed that these taxa are enriched in DS, this should be interpreted as an indication of their ecological presence rather than a definitive measure of their functional activity. To further understand the ecological roles of these taxa, we have complemented the taxonomic analysis with functional predictions using FAPROTAX and BugBase, which suggest that these taxa may contribute to specific stress-related ecological processes in the degraded habitat [64]. Nevertheless, these predictions should be viewed as indicators of potential ecological functions rather than direct evidence of in situ microbial activities.

The beta-diversity analyses reinforce this interpretation by showing that DS formed a clearly separated cluster from RW and MS, indicating that the degraded saline patch harbored the most distinct bacterial assemblage. Given that DS was characterized by the highest pH and EC and the lowest nutrient status, this separation likely reflects strong habitat filtering under severe saline–alkali conditions [65,66]. The phylogenetic distribution of dominant bacterial lineages was broadly consistent with the taxonomic and ordination results. *Pseudomonadota*, *Actinomycetota*, and *Bacteroidota* were more abundant in RW and MS, whereas *Gemmatimonadota*, *Acidobacteriota*, and the RCP2-54 lineage were more strongly associated with DS. Given the broad phylogenetic and metabolic diversity of *Pseudomonadota*, its higher abundance in RW should be interpreted cautiously as a habitat-associated compositional pattern rather than as evidence of a single ecological strategy [67]. Although the present phylogenetic analysis provides a descriptive overview of the relative distribution of dominant bacterial lineages across habitat types, it does not directly test community assembly mechanisms in a formal null-model framework. Therefore, the observed phylogenetic pattern should not be interpreted as evidence of deterministic assembly or stochastic drift, but rather as complementary context for the habitat-associated distribution of dominant taxa [68,69].

The environmental correlation analyses further indicate that the observed bacterial differentiation was closely linked to soil chemical conditions and nutrient-related processes. Mantel, RDA, and envfit analyses consistently highlighted pH, EC, AK, TK, and urease activity as important variables associated with bacterial community variation. Among these, pH and EC likely represent the strongest ecological filters, as both influence microbial physiology, nutrient solubility, osmotic regulation, and enzyme functioning, especially in highly alkaline soils dominated by carbonate and bicarbonate salts [70,71,72,73]. The significant associations of *Pseudomonadota* and *Acidobacteriota* with AK across all samples, together with the significance of TK and URE, indicate that potassium-related variables and nitrogen transformation processes were associated with bacterial community differentiation. However, these relationships should be interpreted as covariation patterns within a broader soil chemical context rather than as evidence that AK directly determined habitat-specific enrichment of individual phyla [74]. In saline–alkali soils, potassium-related variables may reflect not only microbial ion homeostasis and osmotic adjustment, but also underlying soil properties such as mineral composition and cation exchange characteristics [75]. Likewise, urease activity provides a comparative indication of nitrogen transformation potential, but neither AK nor URE alone can establish causal direction in relation to community structure [76,77]. The collective evidence therefore suggests that wetland degradation altered the soil environment in multiple interacting dimensions, and that bacterial community reassembly was associated with the combined effects of salinity–alkalinity stress and nutrient-related constraints rather than with a single dominant factor [78].

The predicted functional and phenotypic results were broadly consistent with the observed structural shifts and provided additional evidence that wetland degradation influenced the ecological characteristics of the bacterial community. FAPROTAX indicated that manganese oxidation and hydrocarbon degradation were more abundant in DS, whereas host- or pathogen-associated functional categories were relatively more abundant in RW and MS. This pattern suggests that the degraded habitat favored bacterial groups associated with stronger oxidation and degradation potential under stressful saline–alkali conditions, while the wetter and more vegetated habitats retained a more complex host-associated ecological context [79]. BugBase prediction showed a similar habitat-dependent pattern at the phenotype level: *Actinomycetota* dominated several predicted phenotypes in RW and MS, whereas *Gemmatimonadota* contributed more strongly to stress-tolerant, Gram-negative, and mobile-element-related phenotypes in DS. Together, these results suggest that degradation not only reshaped bacterial community composition but also shifted the distribution of predicted ecological traits toward stress-associated characteristics in the degraded habitat [80,81].

At the same time, both FAPROTAX and BugBase are taxon-based inference tools, and their outputs should be interpreted as predicted tendencies rather than direct evidence of in situ metabolic activity or phenotype expression [82,83]. In particular, FAPROTAX infers putative ecological functions from taxonomic identities based mainly on cultivated reference strains and therefore cannot fully account for horizontal gene transfer, strain-level variation, or poorly characterized novel lineages. Likewise, BugBase-based phenotype prediction has not been fully validated for complex soil microbial communities and should therefore be interpreted cautiously in non-clinical environmental contexts. Accordingly, the functional and phenotypic patterns reported here should be regarded as predictive ecological signals rather than direct measurements of realized function or phenotype expression. Nevertheless, the agreement among taxonomic reorganization, lineage redistribution, and predicted functional and phenotypic change strengthens the conclusion that wetland degradation in the soda saline–alkali ecosystem was accompanied by coordinated restructuring of belowground bacterial communities [84,85]. In ecological terms, these results suggest that restoring hydrological connectivity, reducing surface salt accumulation, and improving organic matter and nutrient status are important not only for vegetation recovery but also for rebuilding a more stable and functionally balanced soil microbial system [86]. Hydrological restoration can mitigate salt accumulation by enhancing water exchange and reducing evaporative concentration, while also improving soil moisture conditions that favor microbial survival, substrate diffusion, and nutrient turnover [87]. Salt reduction may further relieve osmotic stress and ionic toxicity, thereby weakening the strong environmental filtering imposed on salt-sensitive bacterial groups and facilitating broader community recovery [88]. Improved organic matter and nutrient availability can stimulate microbial metabolism, increase substrate supply, and enhance the functional stability and resilience of the soil microbial community [89]. Therefore, restoration strategies that combine hydrological management, salinity control, and soil quality improvement may help recover not only plant communities but also the microbially mediated ecological functions that support nutrient cycling and ecosystem stability in degraded saline–alkali wetlands [90,91]. Because the present study was based on a single sampling event, future seasonal or longer-term investigations are still needed to evaluate the temporal consistency of these restoration-related microbial responses.

## 5. Conclusions

Wetland degradation in the soda saline–alkali ecosystem markedly altered soil physicochemical properties and enzyme activity, accompanied by clear shifts in bacterial richness, diversity, composition, and community structure. The degraded *Suaeda* habitat showed stronger salinity–alkalinity stress, lower nutrient availability, and a distinct bacterial assemblage enriched in taxa previously reported in saline/alkaline soils, including *Gemmatimonadota*. Environmental variables, especially pH, EC, AK, TK, and urease activity, were closely associated with bacterial community variation, while predicted functional and phenotypic analyses suggested a shift toward stress-related and degradation-associated ecological traits. Taken together, these findings indicate that wetland degradation reshaped the taxonomic composition, phylogenetic distribution, and predicted ecological characteristics of soil bacterial communities in this fragile ecosystem. These inferences were based on 16S rRNA gene data and taxon-based prediction tools, and thus should be regarded as correlational rather than causal. Further validation using higher-resolution omics approaches is still needed. From a restoration perspective, improving hydrological connectivity, reducing salt accumulation, and enhancing soil organic matter and nutrient status may help support the coordinated recovery of vegetation and microbially mediated soil functions in degraded saline–alkali wetlands.

## Figures and Tables

**Table 1:** Alpha diversity indices of soil bacterial communities across different habitat types.

Index	RW	MS	DS	H Statistic	*p*-Value
Sobs	2178.26 ± 479.93 a	2248.57 ± 191.25 a	1033.52 ± 135.47 b	11.47	0.00323
Shannon	5.98 ± 0.41 a	6.09 ± 0.08 a	5.13 ± 0.27 b	9.56	0.00842
Simpson	0.008 ± 0.003 ab	0.006 ± 0.001 b	0.016 ± 0.005 a	9.69	0.00784
Chao 1	2661.94 ± 573.47 a	2766.52 ± 198 a	1211.13 ± 172.92 b	11.41	0.00332

Note: Data are presented as mean ± standard deviation (*n* = 6). Different lowercase letters within a row indicate significant differences among habitat types based on post hoc Tukey–Kramer multiple comparisons. Overall differences among habitat types were evaluated using the Kruskal–Wallis test. RW, reed wetland; MS, meadow steppe; DS, degraded *Suaeda* patch.

## Data Availability

The original contributions presented in this study are included in the article and Supplementary Material. The raw sequencing data have been deposited in the NCBI Sequence Read Archive under BioProject accession number PRJNA1438915.

## Supplementary Materials

The following supporting information can be downloaded at https://www.mdpi.com/article/10.3390/life16050760/s1, Table S1: Envfit analysis of soil environmental variables associated with bacterial community structure along the wetland degradation gradient.

## Abbreviations

The following abbreviations are used in this manuscript:

RW	Reed wetland
MS	Meadow steppe
DS	Degraded *Suaeda* patch
SWC	Soil water content
SOC	Soil organic carbon
TN	Total nitrogen
TP	Total phosphorus
TK	Total potassium
AN	Available nitrogen
AP	Available phosphorus
AK	Available potassium
EC	Electrical conductivity
URE	Urease activity
ALP	Alkaline phosphatase activity
SUC	Sucrase activity
CAT	Catalase activity
OTU	Operational taxonomic unit
PCoA	Principal coordinate analysis
NMDS	Non-metric multidimensional scaling
RDA	Redundancy analysis
LEfSe	Linear discriminant analysis effect size
LDA	Linear discriminant analysis
FDR	False discovery rate

## References

[1] Alikhani S., Nummi P., Ojala A. (2021). Urban Wetlands: A Review on Ecological and Cultural Values. Water.

[2] Herbert E., Boon P., Burgin A., Neubauer S., Franklin R., Ardón M., Hopfensperger K., Lamers L., Gell P. (2015). A global perspective on wetland salinization: Ecological consequences of a growing threat to freshwater wetlands. Ecosphere.

[3] Singh A. (2022). Soil salinity: A global threat to sustainable development. Soil Use Manag..

[4] Zhang Z., Li X., Ren J., Zhou S. (2023). Study on the Drying Process and the Influencing Factors of Desiccation Cracking of Cohesive Soda Saline-Alkali Soil in the Songnen Plain, China. Agriculture.

[5] Pei X., Zhao X., Liu J., Liu W., Zhang H., Jiao J. (2024). Habitat degradation changes and disturbance factors in the Tibetan plateau in the 21st century. Environ. Res..

[6] Zhang J., Zhang Y., Lloyd H., Zhang Z., Li D. (2021). Rapid Reclamation and Degradation of Suaeda salsa Saltmarsh along Coastal China’s Northern Yellow Sea. Land.

[7] Song J., Guan X., Cui H., Liu L., Li Y., Li Y., Ma S. (2025). The impact of salt-tolerant plants on soil nutrients and microbial communities in soda saline-alkali lands of the Songnen plain. Front. Microbiol..

[8] Zhang W., Song T., Su X., Dong W., Yu S. (2026). Wetland degradation changes the sensitivity of soil organic carbon components to exogenous nutrient inputs. Plant Soil.

[9] Liu Q., Jiang S., Wu P., Zhang X., Guo X., Qu Y., Zheng J., Xing Y. (2025). Vegetation-Driven Changes in Soil Properties, Enzymatic Activities, and Microbial Communities of Saline–Alkaline Wetlands. Forests.

[10] Daunoras J., Kačergius A., Gudiukaitė R. (2024). Role of Soil Microbiota Enzymes in Soil Health and Activity Changes Depending on Climate Change and the Type of Soil Ecosystem. Biology.

[11] Zhou X., Tahvanainen T., Malard L., Chen L., Pérez-Pérez J., Berninger F. (2024). Global analysis of soil bacterial genera and diversity in response to pH. Soil Biol. Biochem..

[12] Adetunji A., Lewu F., Mulidzi R., Ncube B. (2017). The biological activities of β-glucosidase, phosphatase and urease as soil quality indicators: A review. J. Soil Sci. Plant Nutr..

[13] Zandi P., Schnug E. (2022). Reactive Oxygen Species, Antioxidant Responses and Implications from a Microbial Modulation Perspective. Biology.

[14] Chang C., Tian L., Tian Z., McLaughlin N., Tian C. (2022). Change of soil microorganism communities under saline-sodic land degradation on the Songnen Plain in northeast China. J. Plant Nutr. Soil Sci..

[15] Hartmann M., Six J. (2023). Soil structure and microbiome functions in agroecosystems. Nat. Rev. Earth Environ..

[16] Rath K., Fierer N., Murphy D., Rousk J. (2018). Linking bacterial community composition to soil salinity along environmental gradients. ISME J..

[17] Bernardin J.R., Young E.B., Cagle G.A., Freedman Z.B., Bittleston L.S. (2025). Environmental Stress Shapes Bacterial Community Structure and Function Through Interactive Abiotic Effects. Mol. Ecol..

[18] Li M., Zhang K., Yan Z., Liu L., Kang E., Kang X. (2022). Soil Water Content Shapes Microbial Community Along Gradients of Wetland Degradation on the Tibetan Plateau. Front. Microbiol..

[19] Xu J., Chen L., Zhou T., Zhang C., Zhang J., Zhao B. (2025). Salinity-driven differentiation of bacterial and fungal communities in coastal wetlands: Contrasting assembly processes and spatial dynamics. Environ. Res..

[20] Bai Z., Jia A., Li H., Wang M., Qu S. (2023). Explore the soil factors driving soil microbial community and structure in Songnen alkaline salt degraded grassland. Front. Plant Sci..

[21] Zhao Z., Song T., Zhang M., Tong S., An Y., Zhang P., Sang B., Cao G. (2023). Benefits of Morphology-Based Functional Group Classification to Study Dynamic Changes in Phytoplankton in Saline-Alkali Wetlands, Taking Typical Saline-Alkali Wetlands in Northeast China as an Example. Diversity.

[22] Wang C., Yu Q., Ji N., Zheng Y., Taylor J., Guo L., Gao C. (2023). Bacterial genome size and gene functional diversity negatively correlate with taxonomic diversity along a pH gradient. Nat. Commun..

[23] Na X., Zang S., Zhang N., Cui J. (2015). Impact of land use and land cover dynamics on Zhalong wetland reserve ecosystem, Heilongjiang Province, China. Int. J. Environ. Sci. Technol..

[24] Wilke B. (2005). Determination of Chemical and Physical Soil Properties. Manual for Soil Analysis—Monitoring and Assessing Soil Bioremediation.

[25] Do D.T., Nguyen H.Q., Do X.S. (2024). Sustainable development in Mekong Delta, Vietnam: Designing resilient cities based on landscape ecological principles. J. Asian Archit. Build. Eng..

[26] Spiteri K., Sacco A. (2024). Estimating the electrical conductivity of a saturated soil paste extract (ECe) from 1:1(EC1:1), 1:2(EC1:2) and 1:5(EC1:5) soil:water suspension ratios, in calcareous soils from the Mediterranean Islands of Malta. Commun. Soil Sci. Plant Anal..

[27] Nóbrega G., Ferreira T., Artur A., Mendonça E., Leão R., Teixeira A., Otero X. (2015). Evaluation of methods for quantifying organic carbon in mangrove soils from semi-arid region. J. Soils Sediments.

[28] Shamrikova E.V., Vanchikova E.V., Kyzyurova E.V., Zhangurov E.V. (2024). Methods for measuring organic carbon content in carbonate-containing soils: A review. Eurasian Soil. Sci..

[29] Zhao G., Sheng Y., Wang J., Li Z., Yang J. (2018). Optimized digestion methods: Organic phosphorus sequential extraction, total phosphorus, and nitrogen simultaneous determination in sediments. J. Soils Sediments.

[30] Banerjee P., Prasad B. (2020). Determination of concentration of total sodium and potassium in surface and ground water using a flame photometer. Appl. Water Sci..

[31] Dodor D., Tabatabai M. (2020). Alkaline Hydrolyzable Organic Nitrogen as an Index of Nitrogen Mineralization in Soils: Relationship with Activities of Arylamidase and Amidohydrolases. Commun. Soil Sci. Plant Anal..

[32] Weligama C., Wasson A., Permalloo G., Delhaize E. (2022). Rapid colorimetric methods for analysis of pH, extractable aluminium and Colwell phosphorus in soils. Soil Res..

[33] Cheng M., Bell R., Brown J., Ma Q., Scanlan C. (2023). Comparison of soil analytical methods for estimating plant-available potassium in highly weathered soils. Soil Res..

[34] Kandeler E., Gerber H. (1988). Short-term assay of soil urease activity using colorimetric determination of ammonium. Biol. Fertil. Soils.

[35] Dou C., Lv Y., Sun Y., Chen X., Li Y. (2024). Assessment of Soil Enzyme Activities in Plant Root Zone of Saline Soil Reclaimed by Drip Irrigation with Saline Groundwater. Agronomy.

[36] Ge Y., Wang Q., Wang L., Liu W., Liu X., Huang Y., Christie P. (2018). Response of soil enzymes and microbial communities to root extracts of the alien Alternanthera philoxeroides. Arch. Agron. Soil Sci..

[37] Abay P., Gong L., Chen X., Luo Y., Wu X. (2022). Spatiotemporal variation and correlation of soil enzyme activities and soil physicochemical properties in canopy gaps of the Tianshan Mountains, Northwest China. J. Arid. Land.

[38] Zeng Q., An S. (2021). Identifying the Biogeographic Patterns of Rare and Abundant Bacterial Communities Using Different Primer Sets on the Loess Plateau. Microorganisms.

[39] Liu J., Xie J., Chu Y., Sun C., Chen C., Wang Q. (2008). Combined effect of cypermethrin and copper on catalase activity in soil. J. Soils Sediments.

[40] Han T.F., Huang J., Liu K.L., Fan H.Z., Shi X.J., Chen J., Jiang X.J., Liu G.R., Zhang L., Xu Y.M. (2021). Soil potassium regulation by changes in potassium balance and iron and aluminum oxides in paddy soils subjected to long-term fertilization regimes. Soil Tillage Res..

[41] Liu J., Zhang H. (2021). Combining multiple markers in environmental DNA metabarcoding to assess deep-sea benthic biodiversity. Front. Mar. Sci..

[42] Roume H., Mondot S., Saliou A., Le Fresne-Languille S., Doré J. (2023). Multicenter evaluation of gut microbiome profiling by next-generation sequencing reveals major biases in partial-length metabarcoding approach. Sci. Rep..

[43] Chen X., Sun H., Jiang F., Shen Y., Li X., Hu X., Shen X., Wei P. (2020). Alteration of the gut microbiota associated with childhood obesity by 16S rRNA gene sequencing. PeerJ.

[44] Corwin D.L., Plant R.E. (2005). Applications of apparent soil electrical conductivity in precision agriculture. Comput. Electron. Agric..

[45] Ding J.N. (2023). Soil nitrogen transformation and functional microbial abundance in an agricultural soil amended with biochar. Rev. Bras. Cienc. Solo.

[46] Schloss P.D., Westcott S.L., Ryabin T., Hall J.R., Hartmann M., Hollister E.B., Weber C.F. (2009). Introducing mothur: Open-source, platform-independent community analysis tools. Appl. Environ. Microbiol..

[47] Han S., Luo X., Hao X., Ouyang Y., Zeng L., Wang L., Wen S., Wang B., Van Nostrand J., Chen W. (2021). Microscale heterogeneity of the soil nitrogen cycling microbial functional structure and potential metabolism. Environ. Microbiol..

[48] Cordero I., Snell H., Bardgett R. (2019). High throughput method for measuring urease activity in soil. Soil Biol. Biochem..

[49] Cui G., Liu Y., Tong S. (2022). Hydrogeochemical processes controlling the salinity of surface water and groundwater in an inland saline-alkali wetland in western Jilin, China. Front. Ecol. Evol..

[50] Hagage M., Abdulaziz A.M., Elbeih S.F., Hewaidy A. (2024). Monitoring soil salinization and waterlogging in the northeastern Nile Delta linked to shallow saline groundwater and irrigation water quality. Sci. Rep..

[51] Ladányi Z., Blanka V., Deák Á., Rakonczai J., Mezősi G. (2016). Assessment of soil and vegetation changes due to hydrologically driven desalinization process in an alkaline wetland, Hungary. Ecol. Complex..

[52] Xu G., Kang X., Wang F., Zhuang W., Yan W., Zhang K. (2024). Alpine wetlands degradation leads to soil nutrient imbalances that affect plant growth and microbial diversity. Commun. Earth Environ..

[53] Liu J., Yu J., Si W., Ding G., Zhang S., Gong D., Bi J. (2023). Variations in bacterial diversity and community structure in the sediments of an alkaline lake in Inner Mongolia plateau, China. PeerJ.

[54] Yang J., Wu X., Ruan H., Song Y., Xu M., Wang S., Wang D., Wu D. (2023). How does grassland degradation affect soil enzyme activity and microbial nutrient limitation in saline–alkaline meadow?. Land Degrad. Dev..

[55] Wang X., Xiao Y., Wang W., Yang X., Zhou G. (2025). Alpine steppe degradation weakens ecosystem multifunctionality through the decline in climax dominant species on the Qinghai-Tibetan plateau. Front. Plant Sci..

[56] Wang K., Mao X., Yang J., Wen M., Han F. (2024). Soil extracellular enzyme activity and microbial resource limitation exhibited close relationships with groundwater table decline in desert wetlands. Catena.

[57] Sui X., Zhang R., Frey B., Yang L., Li M., Ni H. (2019). Land use change effects on diversity of soil bacterial, Acidobacterial and fungal communities in wetlands of the Sanjiang Plain, northeastern China. Sci. Rep..

[58] Chobert S., Roger-Margueritat M., Flandrin L., Berraies S., Lefevre C., Pelosi L., Junier I., Varoquaux N., Pierrel F., Abby S. (2024). Dynamic quinone repertoire accompanied the diversification of energy metabolism in Pseudomonadota. ISME J..

[59] Li C., Li X., Yang Y., Shi Y., Li H. (2022). Degradation reduces the diversity of nitrogen-fixing bacteria in the alpine wetland on the Qinghai-Tibet Plateau. Front. Plant Sci..

[60] Wu Y., Xu N., Wang H., Li J., Zhong H., Dong H., Zeng Z., Zong C. (2021). Variations in the diversity of the soil microbial community and structure under various categories of degraded wetland in Sanjiang Plain, northeastern China. Land Degrad. Dev..

[61] Cui J., Yang B., Zhang M., Song D., Xu X., Ai C., Liang G., Zhou W. (2023). Investigating the effects of organic amendments on soil microbial composition and its linkage to soil organic carbon: A global meta-analysis. Sci. Total Environ..

[62] Li Y., Jiang L., Yuan H., Li E., Yang X. (2024). The Impact of Artificial Afforestation on the Soil Microbial Community and Function in Desertified Areas of NW China. Forests.

[63] Wang H., Chun L., Ji L., Na R., Wei Z., Han W. (2024). Investigating the Diversity and Influencing Factors of the Rhizosphere Bacterial Community Associated with Salicornia europaea L. Populations in Semi-arid Grassland. Agriculture.

[64] Schroeter S., Eveillard D., Chaffron S., Zoppi J., Kampe B., Lohmann P., Jehmlich N., Von Bergen M., Sanchez-Arcos C., Pohnert G. (2022). Microbial community functioning during plant litter decomposition. Sci. Rep..

[65] Ding J., Wang Y., Yu S. (2026). Divergent Assembly of Bacteria and Fungi During Saline–Alkali Wetland Degradation. Biology.

[66] Mucsi M., Borsodi A., Megyes M., Szili-Kovács T. (2024). Response of the metabolic activity and taxonomic composition of bacterial communities to mosaically varying soil salinity and alkalinity. Sci. Rep..

[67] Du J., Wang Z., Hu L., Wang L., Fang J., Liu R. (2024). Comparative Genomics Reveal Distinct Environment Preference and Functional Adaptation Among Lineages of Gemmatimonadota. Microorganisms.

[68] Bourhane Z., Cagnon C., Castañeda C., Rodríguez-Ochoa R., Álvaro-Fuentes J., Cravo-Laureau C., Duran R. (2023). Vertical organization of microbial communities in Salineta hypersaline wetland, Spain. Front. Microbiol..

[69] Dang C., Walkup J., Hungate B., Franklin R., Schwartz E., Morrissey E. (2021). Phylogenetic organization in the assimilation of chemically distinct substrates by soil bacteria. Environ. Microb..

[70] Zhang G., Bai J., Zhai Y., Jia J., Zhao Q., Wang W., Hu X. (2023). Microbial diversity and functions in saline soils: A review from a biogeochemical perspective. J. Adv. Res..

[71] Liu M., Lv X., Zhang W., Jiang M., Tian L., Qin L., Zou Y. (2024). Biological interactions control bacterial but not fungal β diversity during vegetation degradation in saline-alkaline soil. Sci. Total Environ..

[72] Mujakić I., Cabello-Yeves P., Villena-Alemany C., Piwosz K., Rodríguez-Valera F., Picazo A., Camacho A., Koblížek M. (2023). Multi-environment ecogenomics analysis of the cosmopolitan phylum Gemmatimonadota. Microbiol. Spectr..

[73] Feng Y., Guo Z., Zhong L., Zhao F., Zhang J., Lin X. (2017). Balanced Fertilization Decreases Environmental Filtering on Soil Bacterial Community Assemblage in North China. Front. Microbiol..

[74] Wang M., Pu W., Wang S., Zeng X., Sui X., Wang X. (2023). pH-Related Changes in Soil Bacterial Communities in the Sanjiang Plain, Northeast China. Microorganisms.

[75] Barq M., Hassan M., Yasmin H., Shahzad A., Malik N., Lorenz N., Alsahli A., Dick R., Ali N. (2021). Variation in archaeal and bacterial community profiles and their functional metabolic predictions under the influence of pure and mixed fertilizers in paddy soil. Saudi J. Biol. Sci..

[76] Zhu P., Wang Y., Sheng W., Yu M., Wei W., Sun W., Gao J., Xu Z., Cao M., Wang Y. (2025). Salinity Effect on Soil Bacterial and Archaeal Diversity and Assembly in Phragmites australis Salt Marshes in the Qaidam Basin, China. Microorganisms.

[77] Nawaz A., Qamar Z., Marghoob M., Imtiaz M., Imran A., Mubeen F. (2023). Contribution of potassium solubilizing bacteria in improved potassium assimilation and cytosolic K^+^/Na^+^ ratio in rice (*Oryza sativa* L.) under saline-sodic conditions. Front. Microbiol..

[78] Kim J., Roh A., Choi S., Kim E., Choi M., Ahn B., Kim S., Lee Y., Joa J., Kang S. (2016). Soil pH and electrical conductivity are key edaphic factors shaping bacterial communities of greenhouse soils in Korea. J. Microbiol..

[79] Gu Q., Zhang J., Guo W., Wu H., Sun M., Wang J., Wei X., Zhang Y., Chen M., Xue L. (2021). Nitrogen-metabolising microorganism analysis in rapid sand filters from drinking water treatment plant. Environ. Sci. Pollut. Res..

[80] Zhao Q., Bai J., Gao Y., Zhao H., Zhang G., Cui B. (2020). Shifts in the soil bacterial community along a salinity gradient in the Yellow River Delta. Land Degrad. Dev..

[81] Chen S., Zou Y., Zhao C., Liu S., Yu Y., Jiang J., Zou Y., Qiao J. (2024). Study on the Diversity of Bacterial Communities in the Rhizosphere Soils of Different Wild Celery Species in Jilin Province. Agronomy.

[82] Yin F., Zhang F. (2022). Reclamation of abandoned saline-alkali soil increased soil microbial diversity and degradation potential. Plant Soil.

[83] Abulaizi M., Chen M., Yang Z., Hu Y., Zhu X., Jia H. (2023). Response of soil bacterial community to alpine wetland degradation in arid Central Asia. Front. Plant Sci..

[84] Gu Y., Bai Y., Xiang Q., Yu X., Zhao K., Zhang X., Li C., Liu S., Chen Q. (2018). Degradation shaped bacterial and archaeal communities with predictable taxa and their association patterns in Zoige wetland at Tibet plateau. Sci. Rep..

[85] Gong X., Xu L., Langwig M., Chen Z., Huang S., Zhao D., Su L., Zhang Y., Francis C., Liu J. (2024). Globally distributed marine Gemmatimonadota have unique genomic potentials. Microbiome.

[86] Hernandez D., David A., Menges E., Searcy C., Afkhami M. (2021). Environmental stress destabilizes microbial networks. ISME J..

[87] Sansupa C., Wahdan S.F.M., Hossen S., Disayathanoowat T., Wubet T., Purahong W. (2021). Can We Use Functional Annotation of Prokaryotic Taxa (FAPROTAX) to Assign the Ecological Functions of Soil Bacteria?. Appl. Sci..

[88] Langille M.G.I., Zaneveld J., Caporaso J.G., McDonald D., Knights D., Reyes J.A., Clemente J.C., Burkepile D.E., Thurber R.L.V., Knight R. (2013). Predictive Functional Profiling of Microbial Communities Using 16S rRNA Marker Gene Sequences. Nat. Biotechnol..

[89] Coban O., De Deyn G., Van Der Ploeg M. (2022). Soil microbiota as game-changers in restoration of degraded lands. Science.

[90] Zhang G., Bai J., Tebbe C.C., Zhao Q., Jia J., Wang W., Wang X., Yu L. (2021). Salinity controls soil microbial community structure and function in coastal estuarine wetlands. Environ. Microbiol..

[91] Luo S., Yuan J., Song Y., Ren J., Qi J., Zhu M., Feng Y., Li M., Wang B., Li X. (2025). Elevated salinity decreases microbial communities complexity and carbon, nitrogen and phosphorus metabolism in the Songnen Plain wetlands of China. Water. Res..

