# Supplementary material for: Phylogenetic Distribution and Predicted Functional and Ecological Shifts in Soil Bacterial Communities Along a Soda Saline–Alkali Wetland Degradation Gradient
Source: Life (Basel). 2026 May 1;16(5):760. doi: 10.3390/life16050760 (PMC13208205; doi:10.3390/life16050760)
Supplement: Supplementary file 1 [file life-16-00760-s001.zip › life-4265690-supplementary.pdf]

Table S1. Envfit analysis of soil environmental variables associated with bacterial community structure along the wetland degradation gradient

Items	RDA1	RDA2	r^2	*p* value
pH	0.95539	-0.295	0.563	0.004
EC	0.90184	-0.432	0.511	0.003
SWC	-0.99627	0.086	0.074	0.596
SOC	-0.94331	0.332	0.208	0.181
TN	-0.9432	0.332	0.191	0.215
TP	-0.9678	0.252	0.337	0.040
TK	0.99977	0.022	0.258	0.107
AN	-0.94498	0.327	0.228	0.138
AP	-0.96472	0.263	0.205	0.186
AK	-0.94999	0.312	0.429	0.012
CAT	-0.98465	0.175	0.215	0.202
URE	-0.96709	-0.254	0.078	0.537
ALP	0.95605	-0.293	0.489	0.009
SUC	-0.77031	0.638	0.358	0.032

Abbreviations: pH, soil pH value; EC, electrical conductivity; SWC, soil water content; SOC, soil organic carbon; TN, total nitrogen; TP, total phosphorus; TK, total potassium; AN, available nitrogen; AP, available phosphorus; AK, available potassium; URE, soil urease activity; ALP, soil alkaline phosphatase activity; SUC, soil sucrase activity; CAT, soil catalase activity.
